# Supplementary material for: A missense mutation in TUBD1 is associated with high juvenile mortality in Braunvieh and Fleckvieh cattle
Source: BMC Genomics. 2016 May 25;17:400. doi: 10.1186/s12864-016-2742-y (PMC4880872; doi:10.1186/s12864-016-2742-y)
Supplement: Additional file 3: — Genotypes of three sequence variants compatible with recessive inheritance of BH2 in 290 sequenced animals representing seven cattle breeds. The sequenced Braunvieh animals were classified into carrier and non-carrier animals using haplotypes inferred from array-derived genotypes. Blue color indicates the missense mutation in TUBD1. (PDF 34 kb) [file 12864_2016_2742_MOESM3_ESM.pdf]

Position on chromosome 19		11,063,520			11,784,407			11,811,557		
NCBI accession number		rs383232842			rs384588164			NA		
Genotypes		TT	CT	CC	TT	AT	AA	--	A-	AA
Cohort	N									
**Braunvieh**										
BH2-homozygous	1	-	-	1	-	-	1	-	-	1
BH2-heterozygous	5	-	5	-	-	5	-	-	5	-
BH2 non-carrier	48	48	-	-	48	-	-	48	-	-
**Fleckvieh**	149	142	7	-	142	7	-	142	7	-
**Simmental**	15	15	-	-	15	-	-	15	-	-
**Holstein**	51	51	-	-	50	1	-	51	-	-
**Gelbvieh**	12	12	-	-	12	-	-	12	-	-
**Nordic Finncattle**	7	7	-	-	7	-	-	7	-	-
**Ayrshire**	2	2	-	-	2	-	-	2	-	-
