# Supplementary material for: A missense mutation in TUBD1 is associated with high juvenile mortality in Braunvieh and Fleckvieh cattle
Source: BMC Genomics. 2016 May 25;17:400. doi: 10.1186/s12864-016-2742-y (PMC4880872; doi:10.1186/s12864-016-2742-y)
Supplement: Additional file 4: — Across-species conservation of tubulin delta 1. Part of the multispecies alignment of the TUBD1 protein sequence. Blue color highlights the missense mutation. (PDF 73 kb) [file 12864_2016_2742_MOESM4_ESM.pdf]

ENSBTAT00000001700.5: p.H210R

```
Bos taurus                  GEVIVQNYNSILTLSHLYRSSDALLVHENDAVHKICVKLMNIKQVSFCDINQVLAHQLGS
Canis lupus familiaris      GEVIVQNYNSVLTLSHLYRSSDALLVHENDAIHKICAKLMNIKQISFSDINQVLAHQLGS
Homo sapiens                GEVIVQNYNSILTLSHLYRSSDALLLHENDAIHKICAKLMNIKQISFSDINQVLAHQLGS
Macaca fascicularis         GEVIVQNYNSILTLSHLYRSSDALLVHENDAVHKICAKLMNIKQISFSDINQVLAHQLGS
Nannospalax galili          GEVIVQNYNSILTLSHLYRSSDAFLIHENDVVHKICAKRMNIKQISFSNINQVLAHQLGS
Mus musculus                GEVIVQNYNSILTLSHLYRSSDALLIHENDAVHKICAKRMNIKQISFRDLNQVLAHQLGS
Rattus norvegicus           GEVIVQNYNSILTLSHLYRSSDALLIHENDVVHKICAKRMNIKQISFRDLNRVLAHQLGS
Anolis carolinensis         GEVIVQNYNAVLTLSHLYQSSDALLVHENDAVHKICAQLMNIKQISFRDVNRVIAHQLGS
Danio rerio                 GEVIVQNYNSVLTLSHLYQLSDAILVHENDTVHKICSQLMNIKHISISDINKVISHQLAS
Xenopus tropicalis          GEVIVQNYNSILTLSHLYRSSDALLVHENDIIHKVCSQLMNIKQISFRDVNKVIAHQLGS
Gallus gallus               GEVIVQNYNSVLTLSHLYQSSDALLVHENDVIHKICAQLMHIKQISFRDVNQVIAHQLGS
Ciona intestinalis          GEVAVQNYNAILSLSNMCATTDANILLHNNHLHEVCQKLLGLKHVTFTDMNSVAASQLAS
Chlamydomonas reinhardtii   GEVIVQPYNTLLTLSHLADVSDGLVLLENEALHRTAAKLYGIARPSFGVRGRVLGRAGES
                            *** ** **::*:**.:   :*. :: .*: :*. . :   : : ::     * .    *
```

Species	NCBI accession number
Bos taurus	NP_001068938.2
Canis lupus familiaris	NP_001003024.1
Homo sapiens	NP_057345.2
Macaca fascicularis	NP_001272233.1
Nannospalax galili	XP_008840883.1
Mus musculus	NP_001185974.1
Rattus norvegicus	NP_001099296.1
Anolis carolinensis	XP_003227315.1
Danio rerio	NP_001002093.1
Xenopus tropicalis	NP_001006747.1
Gallus gallus	XP_415881.1
Ciona intestinalis	NP_001027643.1
Chlamydomonas reinhardtii	XP_001692014.1
