# Supplementary figures and images for: A missense mutation in TUBD1 is associated with high juvenile mortality in Braunvieh and Fleckvieh cattle
Source: BMC Genomics. 2016 May 25;17:400. doi: 10.1186/s12864-016-2742-y (PMC4880872; doi:10.1186/s12864-016-2742-y)

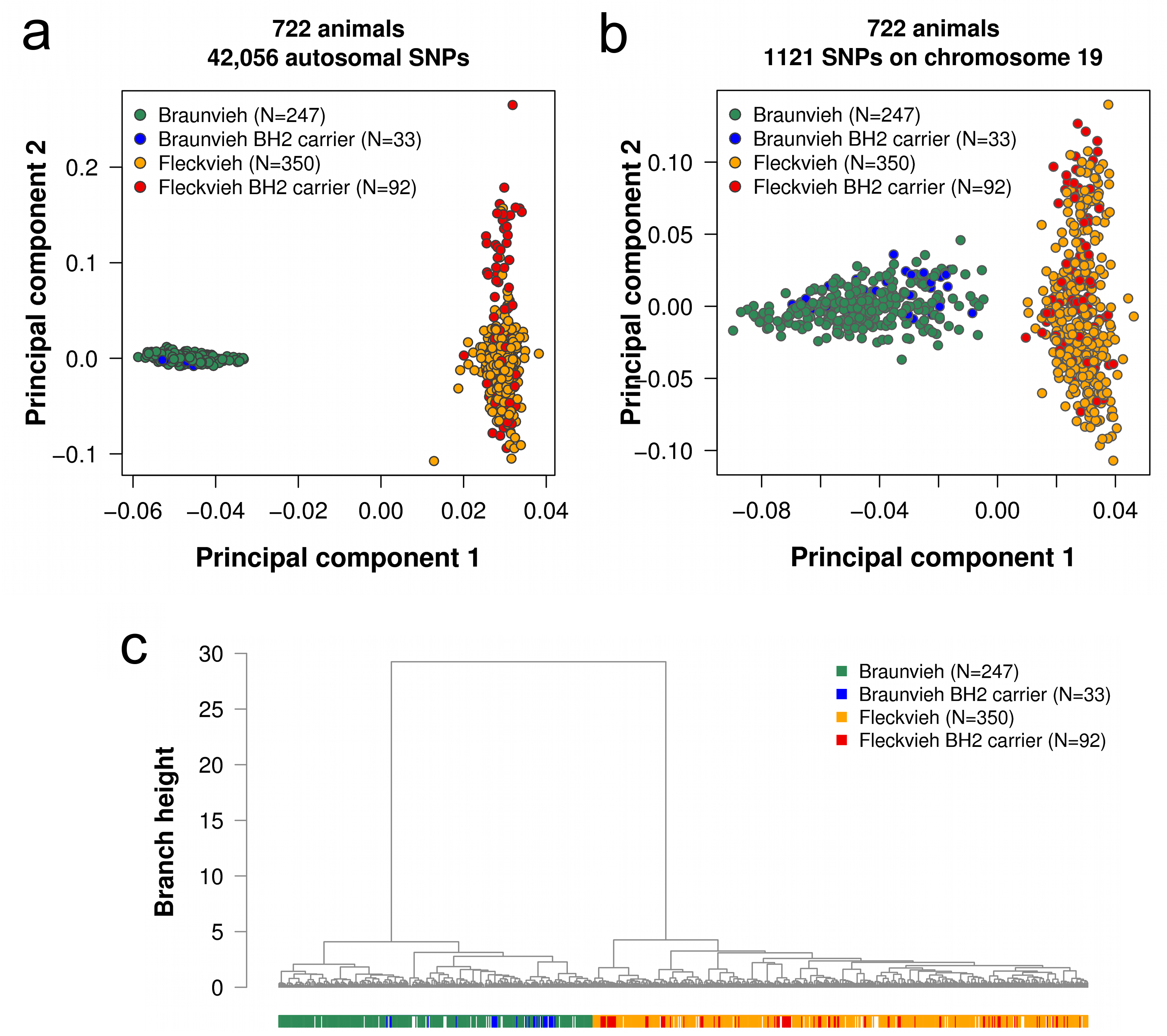

Supplement: Additional file 7: — Cluster analyses in 280 Braunvieh and 442 Fleckvieh animals. Principal component analysis (PCA) using genotypes of 42,056 autosomal SNPs (a) and 1121 SNPs located on chromosome 19 (b), respectively. All animals were born between 1970 and 2010. The PCA separated the animals by breed without any evidence for an admixture. There was no indication that BH2 and BH2FV carriers were more closely related to each other than to non-carrier animals. Hierarchical clustering using genotypes of 1121 SNPs located on chromosome 19 (c). Animals were clearly separated by breed without any evidence of an admixture of BH2 and BH2FV carriers. (TIF 3541 kb) [file 12864_2016_2742_MOESM7_ESM.tif]

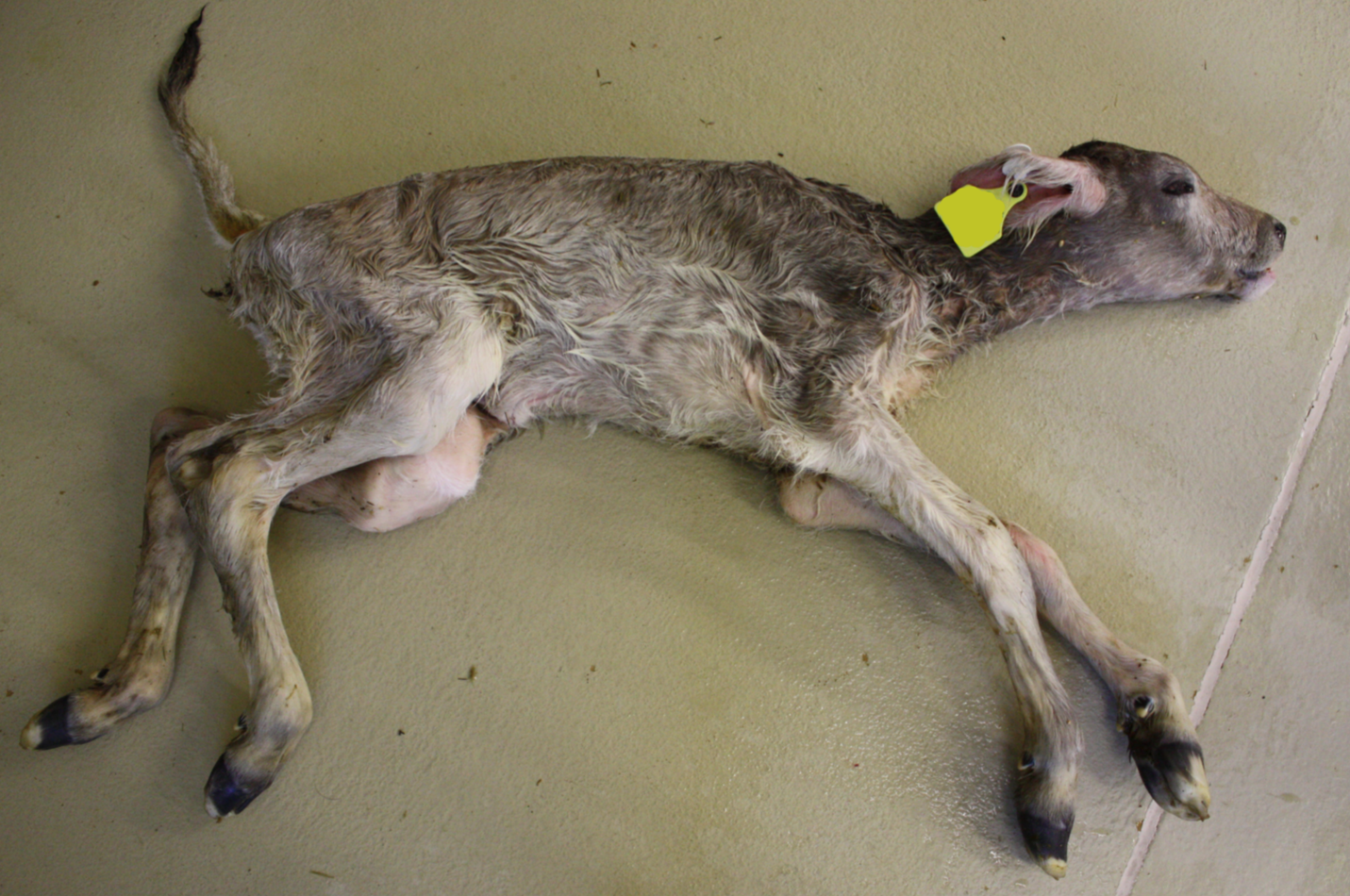

Supplement: Additional file 8: — Photograph of a stillborn homozygous calf. Despite a normal gestation length (283 days), the calf was underweight at birth at 25 kg. The head of the stillborn calf appears elongated and the limbs were thin and elongated. The analysis of histological sections of a diverse tissue panel revealed no pathological findings. (PNG 1867 kb) [file 12864_2016_2742_MOESM8_ESM.png]

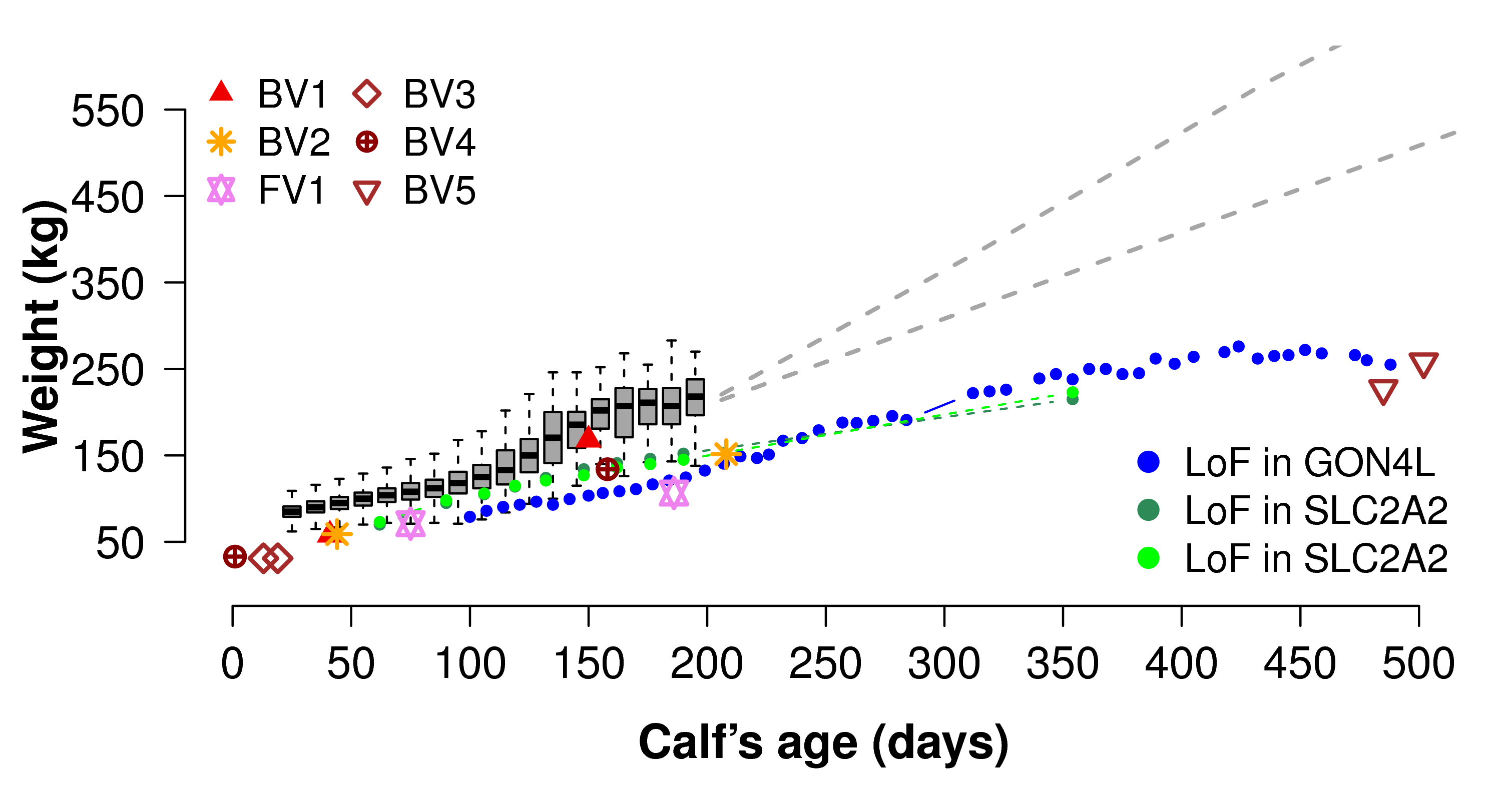

Supplement: Additional file 9: — Growth of six BH2 homozygous calves. The weight of six BH2 homozygous calves (BV1-5, FV1) at admission to the clinic and at euthanasia is compared to 74,422 healthy Fleckvieh animals (grey boxes) and three Fleckvieh animals with loss of function (LoF) variants in GON4L and SLC2A2 that manifest in growth retardation [14, 22]. (TIF 180 kb) [file 12864_2016_2742_MOESM9_ESM.tif]

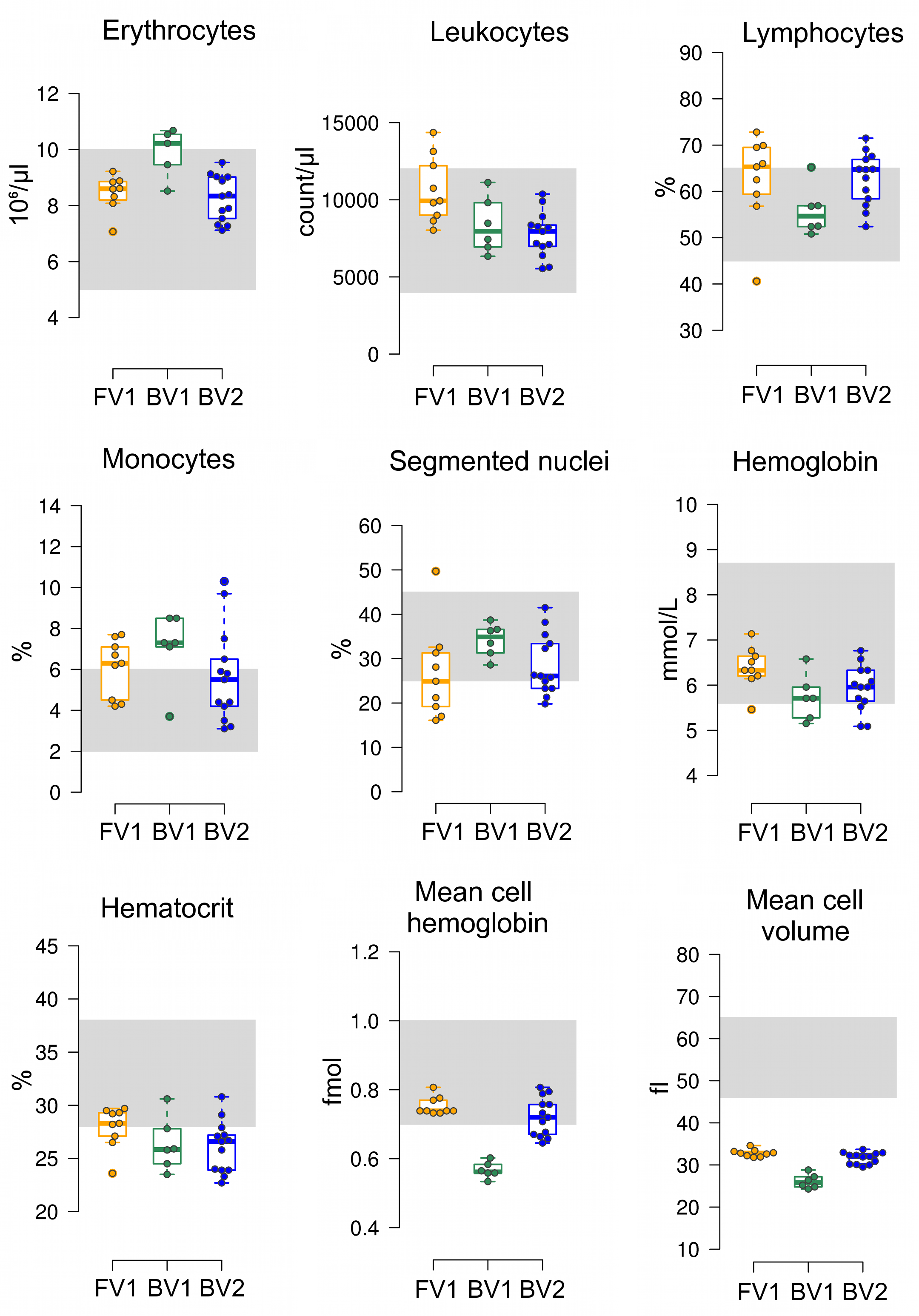

Supplement: Additional file 10: — Blood parameters of BV1, BV2 and FV1. The grey shaded areas represent reference values that were determined based on [69]. (TIF 2443 kb) [file 12864_2016_2742_MOESM10_ESM.tif]

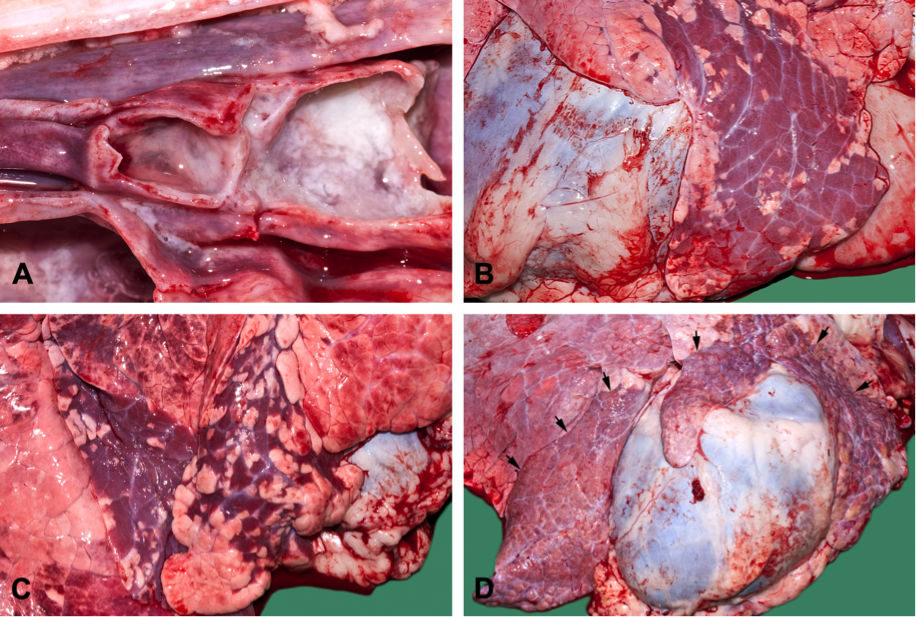

Supplement: Additional file 11: — Macroscopic lesions in the respiratory tract in four homozygous animals. Congestion and mucopurulent inflammation of the nasal conchae of BV2 (a). Different grades of obstruction, atelectasis and/or bronchopneumonia (b-d): single consolidated area in the right cranial lung lobe of BV1 (b), multiple lobular consolidations in both cranial and middle lung lobes of FV1 (c) and severe pneumonic induration of the majority of both cranial and middle lung lobes of BV5 (d). Arrows indicate the border to physiological lung tissue. (PNG 1127 kb) [file 12864_2016_2742_MOESM11_ESM.png]

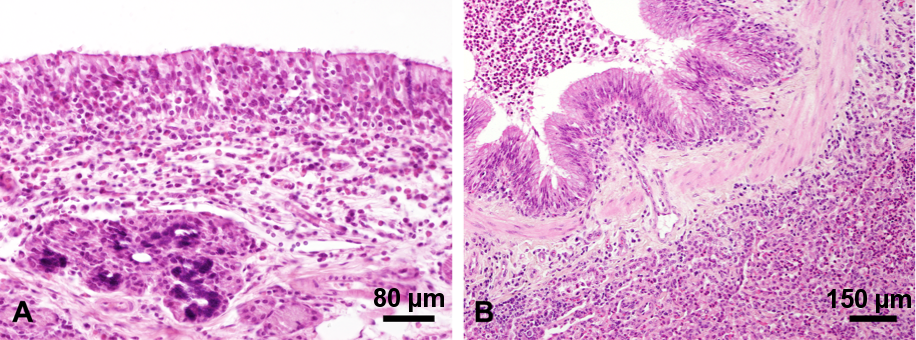

Supplement: Additional file 12: — Histological lesions in the respiratory tract of two homozygous animals. The ciliated epithelium of the nasal mucosa and the underlying submucosa of FV1 are infiltrated with inflammatory cells, particularly neutrophils (a). Lung lesions of BV5 including hyperplastic bronchiolar epithelium, partial obstruction of the bronchiolar lumen by purulent exsudate and consolidation of the surrounding lung tissue due to obstructive atelectasis and mild infiltration with neutrophils (b). (PNG 720 kb) [file 12864_2016_2742_MOESM12_ESM.png]

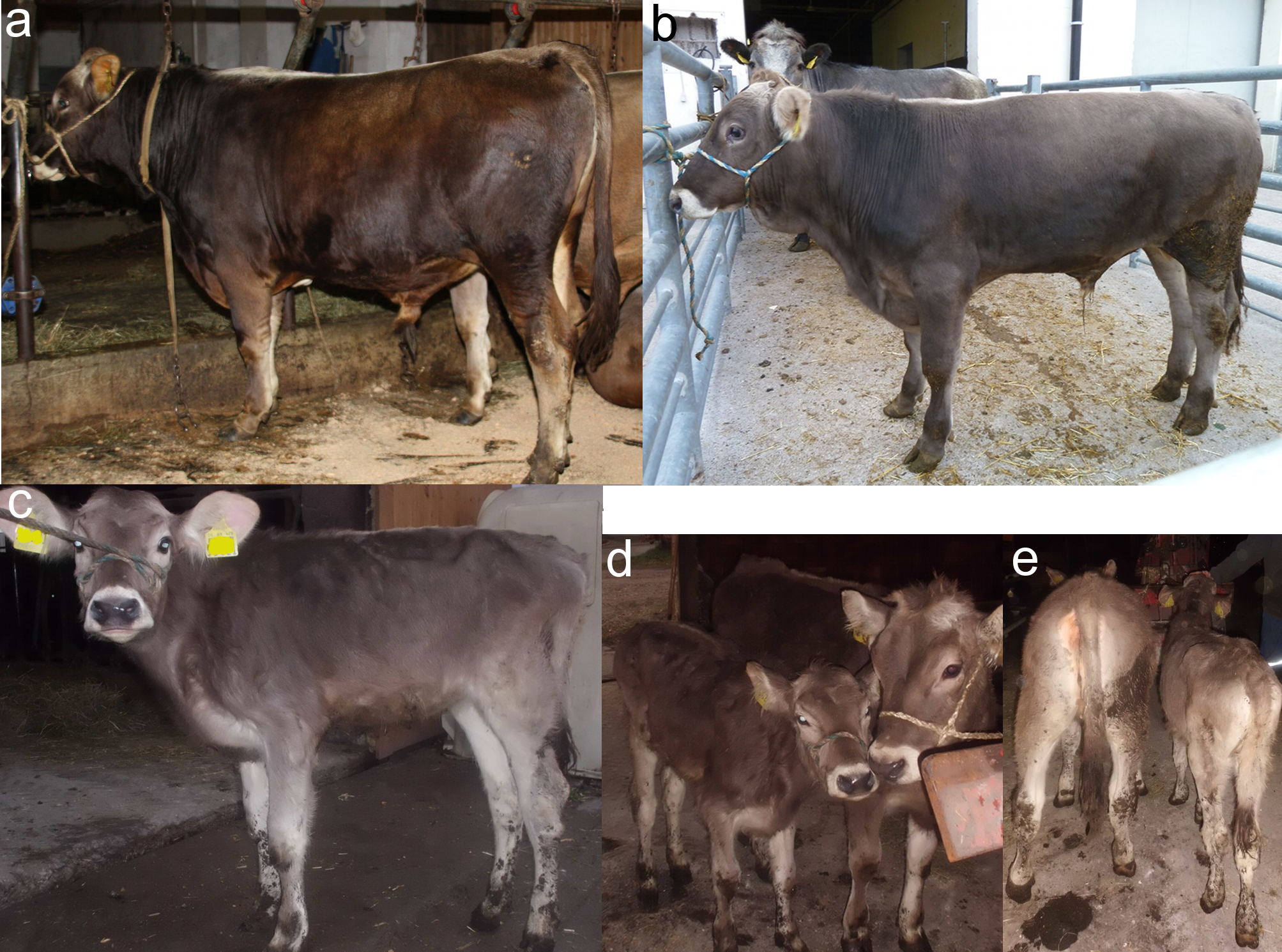

Supplement: Additional file 13: — Three animals homozygous for the rs383232842 C-allele. A homozygous young bull (BV6) at the age of 370 days (a). According to the possessing farmer, the bull did not suffer from respiratory disease in the past. The weight of another homozygous young bull (BV7) at the age of 271 days was 243 kg (b). The possessing farmer reported that the young bull suffered from life-threatening bronchopneumonia early in life. Although the animal recovered from the disease, coughing and excessive mucous exudation from the nostrils were apparent during its entire life. Multiple lung lesions were noticed after slaughter. A female calf homozygous (BV8) for the rs383232842 C-allele at the age of 194 days with an unaffected coeval (c-e). Photographs were kindly provided by Franz Birkenmaier (a, c-e) and Attilio Rossoni (b). (TIF 5396 kb) [file 12864_2016_2742_MOESM13_ESM.tif]
